# Investigating the common genetic basis between inflammatory bowel disease and metabolic syndrome through genomic structural equation modeling

**DOI:** 10.1371/journal.pone.0334456

**Published:** 2025-10-16

**Authors:** Pan Shen, Hao Xiong, Qing-Hua Luo, Yi-Fan Ding, Wei Ge, Wu Liao, Lei-Chang Zhang

**Affiliations:** 1 Clinical Medical College, Jiangxi University of Chinese Medicine, Nanchang, Jiangxi, P.R. China; 2 Department of Anorectal Surgery, Affiliated Hospital of Jiangxi University of Chinese Medicine, Nanchang, Jiangxi, P.R. China; Curtin University Bentley Campus: Curtin University, AUSTRALIA

## Abstract

**Background:**

Inflammatory bowel disease (IBD) and metabolic syndrome (MetS) exhibit a complex interplay, with clinical evidence indicating an increasing incidence of their co-occurrence. However, current research lacks a systematic framework to model the pleiotropic genetic architecture linking gastrointestinal and liver-metabolic phenotypes, thereby hindering a comprehensive understanding of how multiple genetic risk factors converge to drive IBD–MetS comorbidity.

**Methods:**

This study employed genomic structural equation modeling (SEM) to integrate genome-wide association study (GWAS) summary datasets for IBD and MetS-related traits (body mass index, triglycerides, non-alcoholic fatty liver disease, hypertension, and type 2 diabetes), creating the multivariate GWAS summary datasets. Post-GWAS analytical approaches were subsequently utilized to assess risky loci, gene functionality, and tissue-specific regulatory networks, aiming to elucidate the pathological connections between chronic low-grade inflammation and the gut-liver-metabolic axis.

**Results:**

Genomic SEM identified a shared latent genetic factor between IBD and MetS (Comparative Fit Index = 0.9864, Standardized Root Mean Square Residual = 0.0602). A total of 522 lead single nucleotide polymorphism (SNP) loci were identified, including 21 novel SNPs specific to the multivariate model that were not detected in univariate GWAS. Fine-mapping with SuSiE and FINEMAP identified 29 high-confidence causal SNPs. Integrating SNP fine-mapping with MAGMA, FUSION, and FOCUS analyses confirmed seven core genes.

**Conclusion:**

To the best of our knowledge, this study provides the first comprehensive characterization of the shared genetic architecture of IBD and MetS through a multivariate genetic model. The results deepen the understanding of the genetic mechanisms underlying IBD and MetS and offer potential therapeutic targets and a conceptual framework for developing interventions for cross-system diseases.

## 1. Introduction

Inflammatory bowel disease (IBD), primarily encompassing ulcerative colitis and Crohn’s disease, is characterized by chronic, relapsing intestinal inflammation [1,2] and has long been associated with malnutrition [3]. Recent studies have revealed significant comorbid relationships and mechanistic overlaps between IBD and metabolic syndrome (MetS), challenging the traditional view of these as distinct conditions. Increasingly, metabolic disorders, such as obesity, hyperlipidemia, type 2 diabetes (T2D), metabolic dysfunction-associated steatotic liver disease, are being observed in patients with IBD, suggesting a shared inflammation-driven pathogenesis [4,5]. Report indicate a 19.4% prevalence of MetS in patients with IBD [6] with 32% also diagnosed with non-alcoholic fatty liver disease (NAFLD) [5]. Furthermore, Barroso et al. found that elevated abdominal obesity correlates with IBD progression [7]. The gut-liver axis further emphasizes the potential common pathways between IBD and NAFLD [8,9], while a prospective cohort study by Chen et al. linked metabolic dysfunction-associated steatotic liver disease with Crohn’s disease [10].

Despite the clinical distinctions between IBD and MetS, emerging evidence suggests a shared pathological basis, underpinned by low-grade chronic inflammation, metabolic dysfunction, and gut dysbiosis [4]. Recent research has identified associations between IBD and various metabolic conditions, such as obesity [11], hyperlipidemia [12], NAFLD [13], and hypertension (HTN) [14,15]. However, many of these studies predominantly employ bivariate analyses of individual diseases, complicating the identification of true genetic pleiotropy versus false-positive associations driven by phenotypic overlap or diagnostic bias. Moreover, existing analytical models have yet to establish a comprehensive multi-trait genetic framework, limiting our understanding of the shared genetic architecture between IBD and MetS. To address this gap, integrated analyses that consolidate genome-wide association study (GWAS) summary statistics from multiple diseases are essential to identify common genetic factors, thereby overcoming the limitations of traditional single-disease approaches and elucidating the mechanisms behind their comorbidity.

To address these challenges, this study integrated genetic correlations across multiple complex phenotypes to construct a latent genetic model for IBD-MetS comorbidity. Genomic structural equation modeling (SEM) was utilized to analyze GWAS summary statistics for disorders related to IBD and MetS. Genomic SEM facilitates the estimation of shared genetic variation, reflecting common biological processes within a multi-phenotype framework [16,17]. This approach dissects pleiotropy and illuminates the underlying mechanisms driving comorbidity. Although it does not fully encapsulate the intricate relationships between IBD and MetS, it effectively mitigates the confounding effects of established biomarkers and offers novel insights into the genetic architecture of complex disease comorbidities and pleiotropic traits.

To the best of our knowledge, this study represents the first construction of a genomic SEM-based multivariate genetic architecture model within the context of IBD and MetS, aimed at identifying genetic variants with broad effects on both conditions. Emphasizing inflammation as the common pathological driver across gastrointestinal, hepatic, and metabolic systems, the study examined the pathological links between chronic low‐grade inflammation and the gut-liver-metabolic axis. The resulting multivariate structural equation model, termed “multivariate inflammatory gut–liver–metabolic syndrome (mvIGLMS),” was developed to capture these relationships. Following this, various post-GWAS analyses were conducted. These efforts enhance our understanding of the genomic foundation of mvIGLMS and pave the way for further research in this field.

## 2. Methods

**Fig 1:**
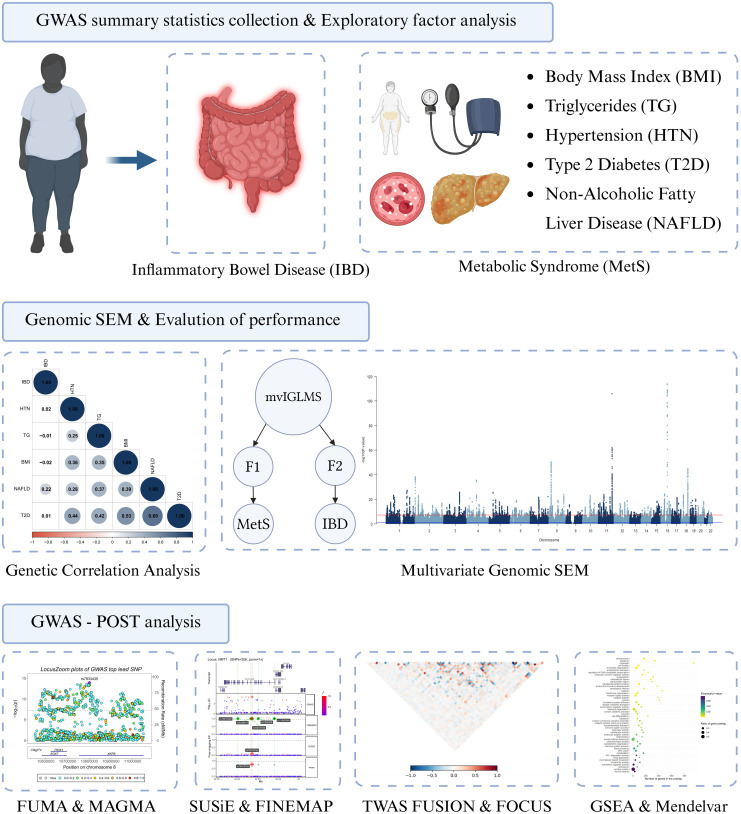
Flowchart illustration of the study design. **Abbreviations:** GWAS = genome-wide association study; SEM = structural equation modelling; mvIGLMS = multivariate inflammatory gut–liver–metabolic syndrome; F1 = first latent factor; F2 = second latent factor; FUMA = Functional Mapping and Annotation; MAGMA = Multi-marker Analysis of GenoMic Annotation; SuSiE = Sum of Single Effects; TWAS = transcriptome-wide association study; FUSION = Functional Summary-based Imputation; FOCUS = Fine-mapping Of CaUsal gene Sets; GSEA = Gene Set Enrichment Analysis.

A flowchart overview is presented in Fig 1.

### 2.1 Sources of univariate GWAS summary statistics

Accurate identification of key phenotypes is crucial for understanding the genetic architecture and mechanisms underlying disease comorbidity. MetS is a multifaceted phenotype rather than a single disease, characterized by several metabolic abnormalities, with central obesity, insulin resistance, dyslipidemia, and hypertension as core features. Building on prior epidemiological and mechanistic studies [18,19], this research selected body mass index (BMI), triglycerides (TG), NAFLD, HTN, and T2D as representative phenotypes. BMI is widely used as a proxy for overall adiposity. Although it does not directly capture central obesity, it is epidemiologically linked to visceral fat accumulation and is therefore relevant to metabolic risk profiling [20]. Elevated TG levels reflect atherogenic dyslipidemia, and BMI, TG, and HTN are essential diagnostic criteria for MetS. T2D, the end stage of glucose metabolic dysregulation, is a critical clinical outcome in MetS progression, including to reflect insulin resistance and glucose metabolism abnormalities. NAFLD, regarded as the hepatic phenotype of MetS, is intricately linked to key MetS pathways, such as insulin resistance, lipotoxicity, and systemic low-grade inflammation, thus encapsulating the syndrome’s multi-organ manifestations [21]. Together, these five phenotypes—BMI, TG, NAFLD, HTN, and T2D—comprehensively represent the physiological and pathological processes central to MetS. Alongside IBD, a mvIGLMS model was constructed.

To ensure the stability and reliability of the model, GWAS summary statistics with the largest sample sizes and SNP counts and the most recent publication dates were prioritized. Only participants of European ancestry were included. Under these criteria, the GWAS summary statistics for “TG” from Richardson et al. were considered most suitable [22]. The GWAS summary statistics for BMI, T2D, and HTN from Elsworth et al. are available through the MRC IEU Open GWAS Project (https://gwas.mrcieu.ac.uk/) [23]. Additionally, GWAS summary statistics for IBD were sourced from the International Inflammatory Bowel Disease Genetics Consortium [24], and those for NAFLD were obtained from the FinnGen database (https://r12.finngen.fi/) [25]. All input GWAS summary statistics were derived from previously published studies with existing ethical approval from the respective institutional review boards, informed consent from participants and rigorous quality control. Detailed information on the GWAS datasets is provided in Table 1.

**Table 1:** Detailed information on the univariate genome-wide association study (GWAS) summary statistics included in the study.

Phenotypes	Phenotypic code	Cases	Controls	N	Prevalence	Ancestry
IBD	ieu-a-31	12882	21770	34652	0.003	European
BMI	ukb-b-19953	NA	NA	461460	NA	European
TG	ieu-b-111	NA	NA	441016	NA	European
NAFLD	finngen_R12_NAFLD	3504	496844	500348	0.324	European
T2D	ukb-b-13806	2972	459961	462933	0.092	European
HTN	ukb-b-14057	119731	343202	462933	0.375	European

**Abbreviations:** N = total sample size; IBD = inflammatory bowel disease; BMI = body mass index; TG = triglycerides; NAFLD = non-alcoholic fatty liver disease; T2D = type 2 diabetes; HTN = hypertension; NA = not applicable.

### 2.2 Sample control for univariate GWAS

The genome reference version used in this study was the Genome Reference Consortium Human Build 37 (GRCh37), corresponding to the UCSC Human Genome version 19 (hg19) assembly. Low-quality data were excluded, and samples with a missing data rate exceeding 5% were removed.

The major histocompatibility complex (MHC) region, located on chromosome (CHR) 6 between approximately 25,000,000 and 35,000,000 base pairs (bp), is characterized by high linkage disequilibrium (LD) [26,27], extensive polymorphism [28], and dense clusters of immune-related genes [29,30]. Due to the genetic diversity and structural complexity of this region—particularly the polymorphisms in immune-related genes—it often generates anomalously strong signals during LD score regression (LDSC) or genomic SEM modeling, which can inflate heritability estimates and destabilize cross-phenotype covariance structures [31]. In our two-factor model, metabolic phenotypes were assigned to the first latent factor (F1), while IBD was exclusively assigned to the second latent factor (F2). Retaining the MHC region could result in an undue dominance of IBD’s genetic signal, potentially distorting F2’s loadings and exaggerating IBD’s explanatory weight, thereby introducing structural noise into factor covariance estimates. Although excluding the MHC region may underestimate the immune-specific genetic contribution to IBD, this trade-off was necessary to preserve the stability of the multivariate model. Accordingly, in line with established practices in genomic SEM analyses, SNPs within this region were excluded from the analysis.

For the mvIGLMS summary statistics, default parameters were applied. All autosomal SNPs from six GWAS related to mvIGLMS were included, filtered according to the recommended quality control criteria, and compared with the 1000 Genomes Phase 3 EUR panel. SNPs with a minor allele frequency (MAF) < 0.01 were excluded, as these are prone to errors due to small sample sizes in genotype clusters and generally exhibit higher standard errors (SE) in LDSC. SNPs with zero effect size estimates were removed to prevent interference with matrix reactivity, which is critical for genomic structural equation modeling. Additionally, SNPs that did not align with the reference panel were excluded, as well as those exhibiting mismatched alleles [32].

### 2.3 Genomic SEM

Genomic SEM was implemented using the genomic SEM R package (version 0.0.5) to perform a genomic structural equation GWAS analysis for IBD, BMI, TG, NAFLD, T2D, and HTN, with the aim of investigating the broad genetic susceptibility underlying these IBD–MetS comorbidity-related traits. Genomic SEM is an advanced multivariate approach that allows exploration of multiple potential models to uncover the underlying structure of traits of interest [16].

To capture the established pathophysiological divergence between IBD and MetS, a two-factor model was constructed. In this model, MetS-related phenotypes (BMI, TG, NAFLD, T2D, and HTN) were assigned to F1, while IBD was assigned to F2. This structure facilitates a clearer distinction between shared and domain-specific genetic influences, thereby avoiding the overgeneralization typically associated with a single common factor model.

Genomic SEM is robust against biases such as sample overlap (e.g., overlapping UKB participants across multiple input GWAS studies) or imbalances in sample sizes. Furthermore, it allows for the identification of variations that affect only a subset of complex traits, rather than all traits, meaning these variations are specific to certain traits and do not represent broad cross-trait susceptibility [33].

The genomic SEM analysis was conducted in two stages. In the first stage, both the empirical genetic covariance matrix and the corresponding sampling covariance matrix were estimated. To this end, summary statistics from the IBD-related and MetS-related GWAS were prepared, and a multivariate extension of cross-trait LDSC version 1.0.1 was applied to generate the empirical genetic covariance matrix for the six traits, which served as the input for the genomic SEM common factor model (S1 Tables [Table 4]). The second stage involved defining the genomic SEM model and minimizing the difference between the hypothesized covariance matrix and the empirical covariance matrix obtained in the first stage. Our primary research objective was to identify the genetic features underlying the six IGLMS-related traits. Consequently, a single-factor model was tested. Model fit was assessed using Standardized Root Mean Square Residual (SRMR), model *χ*^*2*^, Akaike Information Criterion (AIC), and Comparative Fit Index (CFI) (S1 Tables [Table 3]). By applying the appropriate genomic SEM specifications for common factors, individual autosomal SNP associations were integrated into the genetic and sample covariance matrix, generating multi-trait genome-wide analysis results for the structural equation model.

To ensure the robustness of the primary findings, we conducted sensitivity analyses by specifying an alternative multivariate structural equation model for validation. Specifically, due to data availability constraints, the GWAS summary statistics for IBD, BMI, and HTN within the mvIGLMS framework were replaced with alternative datasets (S1 Tables [Table 1]), while maintaining the same quality-control filters as in the main analysis. The genomic SEM framework was then re-fitted, mirroring the primary factor loading structure, and a common-pathway factor GWAS was re-performed. Model stability was further evaluated based on CFI, SRMR, and AIC, and consistency with the primary results was assessed. Post-GWAS analyses were conducted using the same reference panels and parameter settings. Because the substituted datasets included mixed populations, population stratification and LD differences likely reduced overlap at the SNP level. By contrast, gene-level associations, which aggregate signals from multiple variants, showed higher consistency. Sensitivity analyses therefore focused on the gene level, where results were more robust and biologically meaningful.

### 2.4 Genomic SEM SNP heterogeneity

To evaluate the appropriate modeling of SNP associations with mvIGLMS within the multivariate genomic SEM framework, SNP heterogeneity was assessed through statistical analysis. The null hypothesis for the SNP test posits that the SNP’s effect is entirely mediated by the mvIGLMS latent factor across all traits. A significant QSNP test result (P < 0.05) indicates heterogeneity in the SNP’s effects, suggesting that its influence on certain traits is not fully explained by the mvIGLMS latent factor and may operate through alternative mechanisms specific to certain phenotypes. This highlights potential trait-specific genetic pathways [34].

### 2.5 Multi-level evaluation of genomic SEM

The stability of the genomic SEM fit was assessed using the LDSC (version 1.0.1), with parameters including the mean chi-square statistic (*χ*^*2*^), the genomic control inflation factor (*λGC*), the maximum chi-square statistic (*χ*^*2*^*max*), SNP-based heritability (*h*^*2*^), the regression intercept, and the attenuation ratio (calculated as (LDSC intercept – 1)/(mean *χ*^*2*^–1)). No strict quality control filtering was applied to the GWAS summary statistics in this process; SNPs with imputation information score (INFO) < 0.9, MAF < 0.01, or missing entries were retained, as were SNPs with ambiguous strand orientation or anomalous *P*-values. However, partitioned LD scores with zero variance were excluded to prevent instability in the regression estimates.

### 2.6 Defining genomic loci and identifying novel variants

Functional Mapping and Annotation (FUMA) is a web-based platform that annotates, prioritizes, visualizes, and interprets GWAS results. In this study, we used the SNP2GENE module of FUMA (version 1.7.0) to identify genomic risk loci and lead SNPs associated with mvIGLMS. Lead SNPs were defined as variants exhibiting the strongest associations within their respective LD blocks (*r*^*2*^ < 0.1) and achieving genome-wide significance (*P* < 5 × 10^–8^) [35]. A locus was defined by lead SNPs within a 250 kb range and all SNPs in high LD (*r*^*2*^ > 0.6) with at least one independent SNP.

To further assess the novelty of these associations, the lead SNPs and loci were compared with those identified in the original univariate GWAS data, defining a locus was considered novel if it was located more than 1 megabase (Mb, 10⁶ bp) away from previously identified loci in the univariate GWAS summary statistics. To evaluate whether the novel SNPs in mvIGLMS exhibit pleiotropic associations, we examined significant associations (*P* < 5 × 10^–8^) published in the GWAS Catalog.

Furthermore, to identify novel loci emerging only within a multivariate genetic framework, the “GWAS-by-Subtraction” approach was applied. This method compares the lead loci identified by the genomic SEM common factor model with those from univariate GWAS, enabling the discovery of novel, high-utility loci by “subtracting” previously identified signals. These loci, which are undetectable in univariate analyses due to moderate trait-specific effects, may reflect underlying pleiotropic mechanisms.

To functionally characterize the implicated loci at the gene level, the associated output files from FUMA were analyzed using Multi-marker Analysis of GenoMic Annotation (MAGMA). MAGMA is a post-processing tool for GWAS data that evaluates associations between genes and phenotypes (e.g., diseases or health traits) [36]. It integrates multiple genetic markers (such as SNPs) to generate gene-level signals and calculates the association between each gene and the phenotype of interest. The tool extracts gene function-related information from genome-wide SNP data, analyzing genetic signals at the gene level with a false discovery rate (FDR) threshold of 0.05.

### 2.7 SuSiE and FINEMAP analysis to identify causal variants

To identify the most probable causal variants associated with mvIGLMS, Sum of Single Effects (SuSiE) and FINEMAP, implemented in the R package echolocatoR version 2.0.3 [37,38], were applied. A probability threshold of 0.95 was applied to define the credible set of likely causal variants. For each lead SNP that achieved genome-wide significance (*P* < 5 × 10^–8^), a ± 250 kb analysis window centered on the SNP was defined, and fine-mapping was conducted on all SNPs within this window.

SuSiE and FINEMAP calculated the posterior inclusion probability (*PIP*) for each SNP as a potential causal variant and constructed their respective 95% credible sets—subsets of candidate causal SNPs with a cumulative *PIP* of at least 0.95. SNPs included in the credible sets of both methods were classified as consensus causal variants, indicating joint support for their causal potential.

A quantitative measure based on posterior probabilities was further emphasized by calculating the mean *PIP* for each SNP, derived by averaging the *PIP* values from SuSiE and FINEMAP. A threshold of mean *PIP* > 0.5 was used to filter reliable candidate causal variants. Compared to the consensus SNP strategy, integrating mean *PIP* offers a more stable assessment of model consistency, as it captures high causal probability even when a SNP is not present in the credible sets of both tools.

### 2.8 Transcriptome-wide association study (TWAS)

Upon identifying potential causal variants, TWAS analyses was conducted to prioritize genes associated with mvIGLMS based on gene expression-phenotype relationships [39]. The Functional Summary-based Imputation (FUSION) method was utilized for TWAS analyses, incorporating 37,920 pre-calculated expression quantitative trait loci (eQTL) features from the genotype-tissue expression (GTEx) version 8 dataset (i.e., gene-tissue pairs) to compute gene-tissue expression associations [40,41].

Analysis of the TWAS results revealed sufficient variability within mvIGLMS for 36,149 features (out of 37,920 eQTL features), indicating high data quality. Genes with significance (*P* < 0.05) were selected for further analysis.

For genes identified as significant in TWAS, the Fine-mapping Of CaUsal gene Sets (FOCUS, version 0.6.10), a fine-mapping approach designed specifically for TWAS research [42,43], was employed. The FOCUS method evaluates potential causal relationships between genes and phenotypes using *PIP*. Consistent with prior studies, TWAS-significant genes that showed significant associations and aligned with additional evidence from the FOCUS method were further investigated, suggesting their potential causal role.

### 2.9 Gene set and disease ontology enrichment analysis

MAGMA was used to perform a gene-level aggregation analysis of SNP results from genomic SEM, allowing the evaluation of the cumulative gene-level effects of genetic variants associated with the brain-gut comorbidity latent factor [44]. To extend this analysis, the Gene Set Enrichment Analysis (GSEA) module from FUMA (version 1.8.0) was applied for functional enrichment and pathway set analyses on the annotated genes [35,45]. This workflow aimed to identify relevant biological processes, signaling pathways, and potential functional modules related to mvIGLMS. Additionally, MendelVar (https://mendelvar.mrcieu.ac.uk/submit/) was employed to conduct gene enrichment analysis based on mapping genomic loci to known Mendelian disease genes, utilizing lead SNPs to detect pleiotropic genes across phenotypes [46].

### 2.10 Cell annotation analysis

Cell type-specific expression analysis for complex traits was performed using single-cell RNA sequencing data (CELL-type Expression-specific integration for Complex Traits, CELLECT, version 1.3.0) to identify cell types implicated in the etiology of mvIGLMS. The Tabula Muris90 dataset, which includes transcriptomic data from 100,000 cells across 20 organs and tissues of mice (Mus musculus), was used [47]. Single-cell RNA sequencing data were preprocessed and normalized with CELLET, and likelihood scores for the expression specificity of each gene were calculated. Cell-type-specific analysis was performed using LDSC software, classifying cell types based on expression patterns and using a FDR threshold of 0.05 to control for multiple testing [48].

### 2.11 Genetic contribution on genomic regions

The LDSC (version 1.0.1) was used to calculate partitioned heritability across the genome by partitioning genetic variance associated with a phenotype to various genomic regions (such as genes, enhancers, and silencers), to assess the contribution of each region to the overall heritability [49]. LDSC utilizes a weighted LD matrix, genotype frequency files, and summary statistics to estimate the genetic contribution of each region.

## 3. Results

### 3.1 Genomic SEM statistical indicators

Prior to cross-phenotype genetic correlation modeling, LDSC was performed on the six primary study phenotypes (IBD, BMI, TG, NAFLD, T2D, and HTN) to assess the overall strength of their GWAS signals, potential confounding effects, and SNP-level heritability. The LDSC analysis revealed the following heritability contributions for the six univariate GWAS: IBD = 10.5, BMI = 36.1, TG = 13.1, NAFLD = 4.26, T2D = 15, HTN = 23.5. These estimates were statistically significant, supporting the presence of genetic components. Detailed univariate genetic parameters are provided in Table 2, with pairwise genetic covariance values listed in S1 Tables [Table 2].

**Table 2:** LDSC results for each phenotype.

Phenotype	N_SNPs_	*h*^*2*^(SE)	*λGC*	Mean *χ*^*2*^	Intercept (SE)	Ratio (SE)
IBD	1180148	0.3162 (0.0301)	1.175	1.2683	1.0537 (0.0089)	0.2001 (0.0333)
BMI	1174996	0.2481 (0.0069)	2.4611	3.3686	1.1066 (0.022)	0.045 (0.0093)
TG	1111557	0.1504 (0.0115)	1.7665	2.5608	1.1994 (0.053)	0.1278 (0.034)
NAFLD	1160414	0.0051 (0.0012)	1.055	1.0763	1.0256 (0.0079)	0.3354 (0.1041)
T2D	1153594	0.0619 (0.0041)	1.4269	1.5289	1.1861 (0.016)	0.3518 (0.0303)
HTN	1174623	0.111 (0.0047)	1.6941	2.1243	1.0922 (0.0216)	0.082 (0.0192)

**Abbreviations:** LDSC = linkage disequilibrium score regression, version 1.0.1; N_SNPs_ = number of SNPs; *h²* = SNP-based heritability; SE = standard error; *λGC* = genomic control inflation factor; *χ²* = chi-square statistic; Ratio = attenuation ratio; IBD = inflammatory bowel disease; BMI = body mass index; TG = triglycerides; NAFLD = non-alcoholic fatty liver disease; T2D = type 2 diabetes; HTN = hypertension.

To assess potential sample overlap among the included GWAS, LDSC cross-trait intercepts were estimated for each pairwise combination. Most intercepts were close to zero, suggesting limited overlap. However, moderate values were observed between BMI–TG (intercept = 0.3664, SE = 0.0126) and BMI–HTN (intercept = 0.2519, SE = 0.0109), suggesting moderate sample overlap. These values were within the tolerance range of the genomic SEM correction framework and were unlikely to affect overall model stability (cross-trait intercept results are provided in S1 Tables [Table 3]).

**Fig 2:**
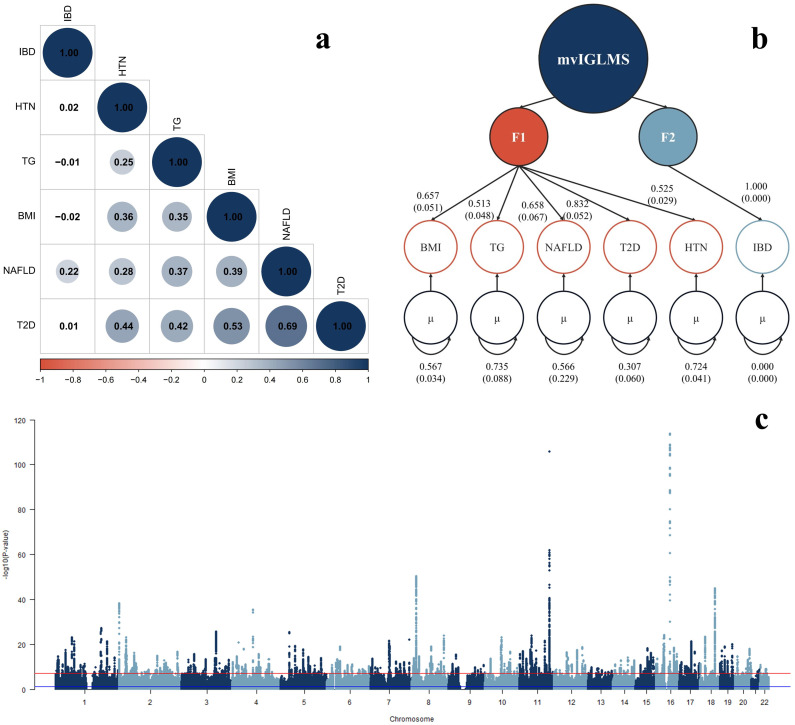
(a) Genetic correlation matrix of inflammatory bowel disease (IBD) and metabolic syndrome (MetS) traits. Circle size, color, and numeric labels represent correlation strength, ranging from −1 to +1. (b) Path diagram of the common factor model estimated by genomic structural equation modeling, showing standardized factor loadings (values in parentheses represent standard errors). The parameter µ represents the residual variance of genetic components that the multivariate inflammatory gut–liver–metabolic syndrome (mvIGLMS) common factor fails to explain in the univariate genome-wide association study summary statistics. (c) Manhattan plot illustrating genome-wide single nucleotide polymorphisms (SNPs) association with mvIGLMS. The y-axis represents −*log*₁₀(*P*), the negative base-10 logarithm of the association *P*-values, where larger values indicate stronger statistical significance. SNPs are ordered by chromosome. The red dashed line denotes the conventional genome-wide significance threshold (*P* = 5 × 10^⁻8^). *P*-values are derived from two-sided Wald tests for each SNP in mvIGLMS. **Abbreviations:** BMI = body mass index; TG = triglycerides; HTN = hypertension; T2D = type 2 diabetes; NAFLD = non-alcoholic fatty liver disease; F1 = first latent factor; F2 = second latent factor.

During model preparation, structural equation modeling analysis was conducted. The genetic covariance matrix for the six univariate GWAS and the empirical covariance matrix fitted well to the common factor model (CFI = 0.9864, SRMR = 0.0602). To enhance transparency, Root Mean Square Error of Approximation (RMSEA) and Tucker–Lewis Index (TLI) were also estimated in lavaan using maximum likelihood estimation with the same covariance input. This analysis yielded a RMSEA of 0.128 and a TLI of 0.843. These discrepancies likely arise from differences in estimation methods: Genomic SEM employs a weighted least squares estimator optimized for LDSC-based summary statistics, while lavaan utilizes classical covariance-based ML estimation, which assumes a homogeneous sample structure. Model stability is summarized in S1 Tables [Table 4a], with parameters for latent factors (F1 and F2) in the univariate structural equation model provided in S1 Tables [Table 4b]. These results offer evidence for the existence of shared genetic factors in mvIGLMS (Fig 2). As a sensitivity analysis, an alternative mvIGLMS model using a partially replaced GWAS dataset also demonstrated acceptable fit indices (AIC = 64.9450, CFI = 0.9739, SRMR = 0.0305), further supporting the robustness of the factor structure.

### 3.2 Genomic SEM GWAS stratified evaluation

Building upon the multivariate genomic SEM framework that integrated IBD and MetS, a GWAS summary statistics dataset for the latent factor representing mvIGLMS was generated, encompassing 6,055,520 SNPs genome-wide.

Further analysis identified 21 novel SNPs that were distinct from those identified in the six univariate GWAS, including those of IBD and MetS. This suggests the increased sensitivity of genomic SEM (Table 3). Notably, several of these novel SNPs overlapped with previously reported GWAS associations related to metabolic traits (e.g., systolic blood pressure, high-density lipoprotein cholesterol, urate levels), inflammation (e.g., eosinophil counts, gamma-glutamyl transferase), and psychiatric and behavioral phenotypes (e.g., mood instability, schizophrenia, smoking, and alcohol consumption). These findings suggest a shared genetic architecture linking inflammation, metabolism, and psychiatric traits. Specifically, these pleiotropic signals highlight the potential mechanistic convergence between IBD and MetS, supporting the hypothesis that systemic inflammatory and metabolic dysregulation may represent a shared underlying mechanism.

**Table 3:** Novel single nucleotide polymorphisms (SNPs) identified in genomic structural equation modelling (SEM) analysis for multivariate inflammatory gut–liver–metabolic syndrome (mvIGLMS).

SNP	CHR	BP	NEA	EA	MAF	*P*	BETA	SE	Nearest gene
rs10930993	2	183084726	C	A	0.170	7.82 × 10^−9^	0.005	0.0009	*PDE1A*
rs11064885	12	110498432	A	C	0.103	4.72 × 10^−8^	0.006	0.0011	*C12orf76*
rs11531420	5	148069705	C	T	0.242	3.36 × 10^−8^	0.004	0.0008	*HTR4*
rs12142313	1	198772260	G	T	0.150	1.63 × 10^−10^	−0.006	0.0009	*MIR181A1HG*
rs12801000	11	57080352	A	G	0.045	4.68 × 10^−9^	0.009	0.0016	*TNKS1 BP1*
rs136832	22	40046538	T	C	0.211	7.91 × 10^−9^	−0.005	0.0008	*CACNA1I*
rs137863610	10	75049711	A	G	0.037	4.72 × 10^−8^	0.010	0.0018	*CFAP70* ^†^
rs1985524	5	147847788	C	G	0.406	8.98 × 10^−9^	0.004	0.0007	*HTR4*
rs2238071	12	2456416	G	A	0.458	1.50 × 10^−8^	−0.004	0.0007	*CACNA1C*
rs2369490	12	897564	C	G	0.297	3.91 × 10^−8^	−0.004	0.0007	*WNK1*
rs2846050	11	57039870	C	T	0.308	1.82 × 10^−8^	0.004	0.0007	*TNKS1 BP1*
rs34224566	17	79382416	T	C	0.247	3.39 × 10^−8^	−0.004	0.0007	*RP11-1055B8.7* ^‡^
rs35797471	14	104621383	T	C	0.464	4.02 × 10^−8^	−0.004	0.0007	*KIF26A*
rs4879799	9	34509526	A	G	0.361	1.44 × 10^−8^	0.004	0.0007	*DNAI1*
rs6087845	20	30832433	C	G	0.156	2.94 × 10^−8^	−0.005	0.0009	*POFUT1*
rs61596977	8	95997165	T	C	0.127	2.98 × 10^−8^	−0.005	0.0009	*NDUFAF6*
rs62190671	2	162652961	G	A	0.100	9.83 × 10^−9^	0.007	0.0012	*SLC4A10*
rs634366	11	103020971	C	T	0.259	1.13 × 10^−8^	−0.004	0.0007	*DYNC2H1*
rs7612014	3	167467135	G	A	0.194	1.24 × 10^−8^	−0.004	0.0008	*SERPINI1*
rs8050566	16	3611610	T	C	0.435	7.22 × 10^-11^	−0.004	0.0007	*NLRC3*
rs997123	2	142852502	C	T	0.485	2.15 × 10^−8^	0.004	0.0007	*LRP1B*

**Abbreviations:** CHR = chromosome; BP = base pair position; NEA = non-effect allele; EA = effect allele; MAF = minor allele frequency; *P* = *P*-value; BETA = regression coefficient (effect size); SE = standard error.

Gene symbols follow HUGO Gene Nomenclature Committee (HGNC)-approved nomenclature. † *CFAP70* = approved symbol, formerly *TTC18*. ‡ *RP11-1055B8.7* = uncharacterized lncRNA, Ensembl annotation, not HGNC-approved.

### 3.3 Genomic SEM evaluation based on LDSC genomic control

After applying parameter control within the genomic SEM framework, 4,989,906 SNPs were excluded, leaving 1,065,614 SNPs retained for regression analysis. The mean *χ*^*2*^ across all SNPs was 2.466, with a *λGC* of 1.908. The *χ*^*2*^max was 516.401, and 6,109 SNPs surpassed the genome-wide significance threshold. The heterogeneity test revealed no significant heterogeneity (*P* > 0.05). The estimated heritability on the observed scale was *h*^*2*^ = 0.197 (SE = 0.006), with the genetic-to-environmental contribution ratio estimated at 0.005 (SE = 0.012). The regression intercept was 1.007 (SE = 0.017). These results suggest that the observed inflation in the structural equation model is likely attributable to polygenic heritability signals rather than population stratification or pleiotropy.

### 3.4 Evaluation of mvIGLMS genomic SEM using FUMA software

**Fig 3:**
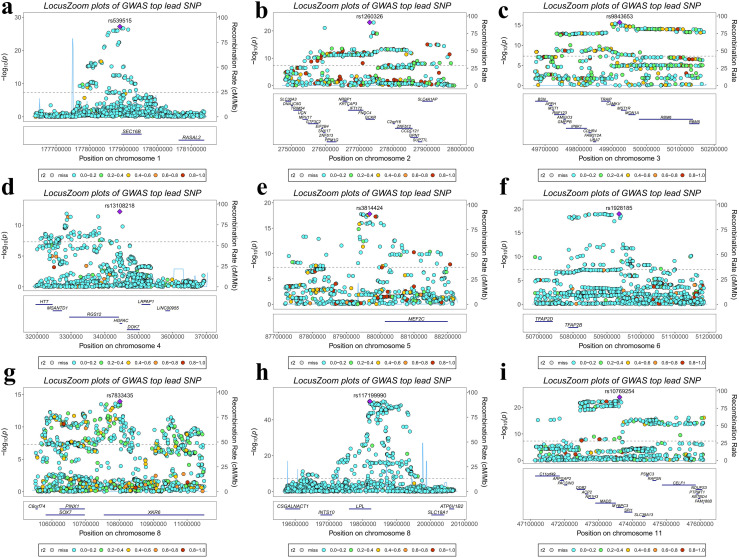
Regional association plots for representative risk loci identified by genomic structural equation modeling of multivariate inflammatory gut–liver–metabolic syndrome (mvIGLMS). LocusZoom plots show regional association signals surrounding the top lead single nucleotide polymorphisms (SNPs) (purple diamonds) for nine representative risk loci (a–i) prioritized by Functional Mapping and Annotation (FUMA) based on the mvIGLMS. The x-axis denotes the genomic position, and the y-axis indicates –*log*₁₀(*P*) of SNP associations. Variants are colored by linkage disequilibrium (*r²*) with the lead SNP, as shown in the legend. Gene annotations are shown below each panel, and horizontal dashed lines indicate the genome-wide significance threshold (*P* = 5 × 10 ⁻ ⁸). Loci were selected for display based on a combination of statistical significance, linkage disequilibrium structure, gene annotation density, and biological interpretability, aiming to showcase representative and mechanistically relevant regions. **Abbreviations:** GWAS = genome-wide association study.

Using FUMA, the genomic SEM framework for mvIGLMS was evaluated, identifying 356 genomic risk loci (detailed risk loci are presented in S1 Tables [Table 5], Fig 3). Additionally, MAGMA identified 895 candidate genes potentially associated with mvIGLMS, applying a significance threshold of *P* < 2.732 × 10^–6^ (Bonferroni correction, 0.05/18,304) (A total of 622 genes were significantly associated in both the primary and sensitivity analyses, and detailed MAGMA results are presented in S1 Tables [Table 6])*.* Through FUMA, 522 lead SNPs were annotated, with most located in intronic regions, while others mapped to exonic, untranslated regions (UTRs), regulatory, or intergenic regions (detailed lead SNPs are presented in S1 Tables [Table 7]). S1 Tables [Table 8] further details the effect sizes, directions, and significance levels of each lead SNP across multiple phenotypes, highlighting their pleiotropic or phenotype-specific associations. This integration of cross-phenotype data helps uncover shared genetic architectures across diverse phenotypes and assess whether specific risk loci exert selective effects on particular traits.

### 3.5 SuSiE and FINEMAP analysis reveals potential causal variants for mvIGLMS

To identify the potential causal genetic variants associated with the mvIGLMS factor, analyses were conducted using SuSiE and FINEMAP, identifying 112 SNPs with mean *PIP* > 0.5, which were considered variants with moderate confidence (detailed SuSiE and FINEMAP results are presented in S1 Tables [Table 9]).

**Fig 4:**
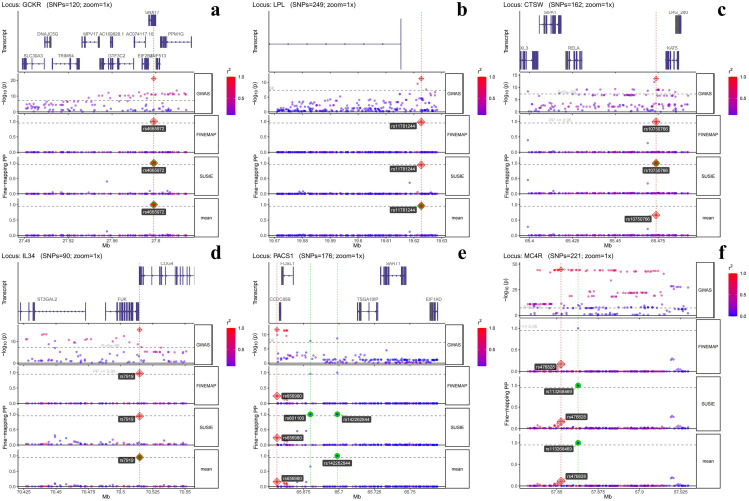
Fine-mapping results for representative loci associated with the multivariate inflammatory gut–liver–metabolic syndrome (mvIGLMS). Panels (a–f) display locus-specific views for *GCKR*, *LPL*, *CTSW*, *IL34*, *PACS1*, and *MC4R*, respectively. Loci were selected for display based on high mean posterior inclusion probabilities (*PIP*, noted as “*PP*” in figure panels) (> 0.95), strong local linkage disequilibrium structure, dense regional gene annotations, and the presence of multiple credible single nucleotide polymorphisms (SNPs). For each locus, tracks show: (i) gene models; (ii) genome-wide association study (GWAS) association signals (The y-axis represents –*log*₁₀[*P*]); (iii) *PIP* from FINEMAP; (iv) *PIP* from Sum of Single Effects (SUSiE); and (v) the mean *PIP* across the two methods. The x-axis represents chromosomal position (in megabase [Mb]); y-axis represent –*log*₁₀(*P*) or *PIP*. Lead SNPs are highlighted with vertical dashed lines and are color-coded by linkage disequilibrium (*r²*) with the top signal.

Variants with a mean *PIP* > 0.95 were prioritized as high-confidence candidate causal loci, supported by both models. After screening, 29 SNPs met this stringent criterion and were located in the following gene-proximal regions: Chromosome 2: rs2305929 (*BABAM2*, formerly *BRE*), rs4665972 (*GCKR*), rs62106258 (*TMEM18*), rs72770995 (*SH3YL1*), rs77730916 (*PANTR1*, formerly *LINC01158*); Chromosome 3: rs7638388 (*MAP4*), rs9839992 (*ARPP21*), rs115002913 (*CACNA1D*); Chromosome 7: rs73044453 (*ZFAND2A*); Chromosome 8: rs2943611 (*AC016194.1,* uncharacterized lncRNA, Ensembl annotation; same below for *RP11/AC* loci), rs11781244 (*LPL*); Chromosome 10: rs72822049 (*RP11-491H19.1*); Chromosome 11: rs142262844 (*PACS1*), rs17474890 (*RP11-347H15.1*), rs67257108 (*SBF2*); Chromosome 12: rs7953257 (*PTPN11*), rs74609289 (*CUX2*), rs12370007 (*HOXC4*); Chromosome 14: rs113644913 (*KIF26A*); Chromosome 15: rs4778599 (*ABHD17C*); Chromosome 16: rs62055819 and rs79998024 (*UTP4*, formerly *CIRH1A*), rs34366687 (*PKD1L3*), rs7919 (*IL34*); Chromosome 17: rs72835405 (*RP11-81K2.1* and *RP11-1079K10.2*); Chromosome 18: rs34633411 (*RP11-325K19.3*), rs113268469 (*MC4R*); Chromosome 20: rs2427538 (*OPRL1*). The regional genomic profiles showed clear association peaks at these loci (Fig 4).

### 3.6 TWAS analysis and fine-mapping to identify high-confidence functional genes

**Fig 5:**
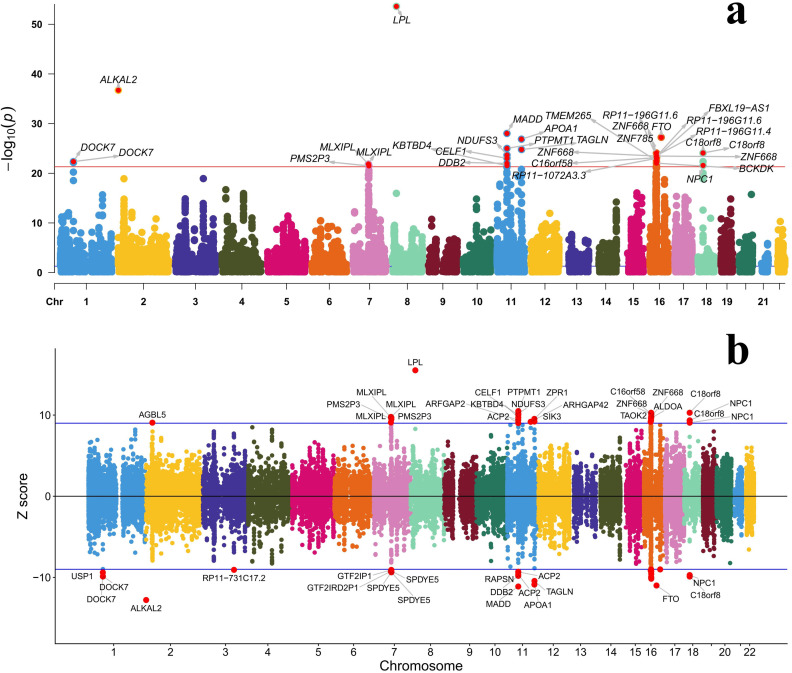
Manhattan plot of transcriptome-wide association study (TWAS) results for the multivariate inflammatory gut–liver–metabolic syndrome (mvIGLMS) derived using sparse canonical correlation analysis (sCCA). (a) Manhattan plot showing gene-based associations across chromosomes. The y-axis represents −*log*_10_(*P*), the negative base-10 logarithm of the TWAS *P*-values. The horizontal red line corresponds to the TWAS significance threshold of *P* = 5 × 10^⁻22^. (b) Manhattan plot of mvIGLMS TWAS *Z*-scores across chromosomes. The y-axis shows standardized test statistics (*Z*-scores). The horizontal blue line indicates |*Z*| = 9.

TWAS analysis was performed on the GWAS summary statistics for mvIGLMS using the FUSION framework, with a significance threshold set at *P* < 1.318 × 10^–6^ (Bonferroni correction, 0.05/ 37,920). A total of 1,191 genes exceeded this threshold (detailed TWAS FUSION results are presented in S1 Tables [Table 10a], Fig 5). Fine-mapping analysis was subsequently conducted using FOCUS, which computed the *PIP* for each candidate gene. Of the 871 genes exhibiting a *PIP* > 0.50, 360 demonstrated high-confidence functional associations for mvIGLMS (*PIP* > 0.95) (detailed FOCUS results are presented in S1 Tables [Table 10b]).

To further validate these “high-confidence” gene-level associations, an overlap analysis was conducted. High-confidence genes were defined as those meeting both criteria: *PIP* > 0.95 and a significant TWAS *P*-value (Bonferroni corrected, *P* < 0.05/37,920). Notably, the TWAS *Z*-scores for genes such as *AGBL5*, *MLXIPL*, *PMS2P3*, *LPL*, *ARFGAP2*, *CELF1*, *PTPMT1*, *ZPR1*, *NPC1*, *KBTBD4*, *ACP2*, *NDUFS3*, *SIK3*, *ARHGAP42*, *ZNF668*, *TAOK2*, *ALDOA*, *C18orf8*, *NPC1*, and others had positive TWAS *Z*-scores, indicating a positive correlation between predicted gene expression and mvIGLMS. This suggests that higher expression of these genes may increase susceptibility to mvIGLMS. Conversely, TWAS *Z*-scores for genes such as *USP1*, *DOCK7*, *ALKAL2*, *RP11*-*731C17.2*, *GTF2IP1*, *GTF2IRD2P1*, *SPDYE5*, *RAPSN*, *DDB2*, *MADD*, *ACP2*, *APOA1*, *TAGLN*, *FTO*, *NPC1*, and others were less than 0, indicating that reduced predicted expression of these genes may be associated with increased risk of mvIGLMS (refer to S1 Tables [Table 10c] for the TWAS and FOCUS intersection results).

### 3.7 Gene intersection test

Based on the aforementioned analyses, an intersection analysis was conducted on the gene sets identified by MAGMA, SuSiE, FINEMAP, FUSION, and FOCUS. Genes were filtered using the following criteria: a significance threshold of *P* < 0.05 (FDR corrected) for the MAGMA method, a mean *PIP* > 0.5 for the SuSiE and FINEMAP methods, *P* < 1.318 × 10^–6^ (Bonferroni corrected, 0.05/37,920) for the FUSION method, and a *PIP* > 0.95 for the FOCUS method. The results suggested that *BABAM2 (BRISC and BRCA1 A complex member 2,* formerly *BRE)*, *ABHD17C (abhydrolase domain containing 17C, depalmitoylase)*, *SH3YL1 (SH3 and SYLF domain containing 1)*, *HOXC4 (homeobox C4)*, *LPL (lipoprotein lipase)*, *NMT1 (N-myristoyltransferase 1)*, and *CTSW (cathepsin W)* were consistently identified as high-confidence genes across multiple analytical approaches (Table 4 displays the gene-level results from the different methods). All seven high-confidence genes (*BABAM2*, *ABHD17C*, *SH3YL1*, *HOXC4*, *LPL*, *NMT1*, and *CTSW*) also passed the sensitivity analyses validation, reaching the same significance thresholds at the gene level (S1 Tables [Table 11]).

**Table 4:** High-confidence candidate genes prioritized by integrative analysis across MAGMA, SuSiE, FINEMAP, FUSION, and FOCUS.

Gene	SNP	Mean *PIP*	*P*_FUSION_	*PIP*_FOCUS_	P_FDR_ (MAGMA)
*BABAM2* ^†^	rs2305929	1.000	1.35 × 10^-11^	1.000	2.27 × 10^−4^
*ABHD17C*	rs4778599	0.990	8.79 × 10^-16^	1.000	9.18 × 10^−6^
*SH3YL1*	rs72770995	0.985	8.91 × 10^−8^	1.000	4.18 × 10^−3^
*HOXC4*	rs12370007	0.982	4.81 × 10^−10^	1.000	1.90 × 10^−9^
*LPL*	rs11781244	0.961	2.59 × 10^-54^	1.000	9.18 × 10^−6^
*NMT1*	rs74815784	0.772	1.72 × 10^−8^	0.999	5.26 × 10^−4^
*NMT1*	rs75810445	0.756	1.72 × 10^−8^	0.999	5.26 × 10^−4^
*NMT1*	rs2304988	0.750	1.72 × 10^−8^	0.999	5.26 × 10^−4^
*NMT1*	rs11650246	0.750	1.72 × 10^−8^	0.999	5.26 × 10^−4^
*NMT1*	rs2289674	0.750	1.72 × 10^−8^	0.999	5.26 × 10^−4^
*CTSW*	rs10750766	0.662	1.99 × 10^-12^	0.988	6.78 × 10^−6^

**Abbreviations:** MAGMA = Multi-marker Analysis of GenoMic Annotation; SuSiE = Sum of Single Effects; FUSION = Functional Summary-based Imputation; FOCUS = Fine-mapping Of CaUsal gene Sets; SNP = single nucleotide polymorphism; mean *PIP* = mean posterior inclusion probability from SuSiE and FINEMAP; *P*_FUSION_ = *P*-value from transcriptome-wide association study (TWAS) using FUSION; *PIP*_FOCUS_ = posterior inclusion probability from FOCUS; *P*_FDR_ (MAGMA) = false discovery rate (FDR)-adjusted *P*-value from MAGMA gene-level association analysis.

† *BABAM2 = *approved symbol, formerly *BRE.*

### 3.8 Pathway and cell type enrichment and mendelian gene enrichment

A gene-level annotation and aggregation analysis of the GWAS summary statistics for mvIGLMS was conducted using MAGMA, identifying 895 significantly associated genes (detailed MAGMA results are presented in S1 Tables [Table 6]). Based on this gene set, GSEA was performed. In addition to the expected enrichment in metabolic and inflammatory pathways, several additional pathways exhibited significant enrichment. Gene sets related to brain morphology (adjusted *P* [adj*P*] = 1.69 × 10^–55^), cognitive function (e.g., extremely high intelligence, adj*P* = 4.11 × 10^–27^), and psychiatric disorders such as schizophrenia and bipolar disorder (adj*P* = 1 × 10^–17^) were significantly enriched, involving core genes critical for neurodevelopment, synaptic function, and cognitive regulation (e.g., *BDNF*, *MEF2C*, *NRXN3*, *CACNA1C*). These results suggest that the comorbidity between IBD and MetS may be partially mediated through the brain–gut–metabolic axis. Moreover, several gene-environment interaction (G × E) pathways related to lifestyle factors showed statistically significant enrichment, such as interactions between diastolic blood pressure and alcohol consumption, triglycerides and alcohol consumption, and systolic blood pressure and smoking status (adj*P* values ranging from 1.0 × 10^–38^ to 1.0 × 10^–15^). These findings indicate that certain susceptibility loci may have enhanced effects under specific environmental exposures, indicating behavior- and environment- dependent genetic mechanisms underlying IBD-MetS comorbidity. Notably, chromosomal locus gene set analysis revealed significant enrichment in the chr16p11 and chr11p11 regions, with adj*P* values of 5.46 × 10^–52^ and 9.95 × 10^–30^, respectively. These regions encompass several putative regulatory genes (e.g., *SH2B1*, *TAOK2*, *ATP2A1*, *FES*), which may represent previously uncharacterized regulatory hotspots for comorbidity (detailed GSEA results are presented in S1 Tables [Table 12]).

**Fig 6:**
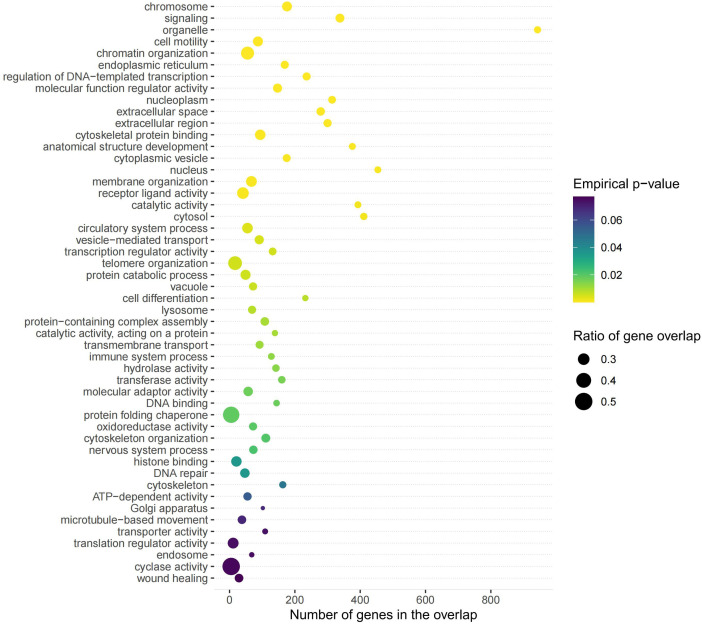
Gene ontology enrichment results based on MendelVar analysis of multivariate inflammatory gut–liver–metabolic syndrome (mvIGLMS)-associated loci. Each bubble represents a specific disease and its associated gene overlap along with its statistical significance. The x-axis denotes the number of genes overlapping each ontology term. The y-axis lists enriched gene ontology (GO) categories. Bubble size reflects the ratio of overlapping genes relative to the total number of genes annotated to each term, and bubble color indicates *P*-values (yellow to purple), with more significant terms shown in yellow.

MendelVar analysis indicated that genes associated with mvIGLMS were significantly enriched in several Gene Ontology terms related to immune function and inflammatory response, including “immune system process,” “lysosome,” and “hydrolase activity.” This observation suggests that these genes may contribute to the comorbidity of IBD and MetS by modulating processes such as innate immune activation, antigen processing, and inflammatory mediator metabolism. Simultaneously, a subset of these genes was also concentrated in pathways intimately linked with organelle homeostasis and energy metabolism, such as those associated with “endoplasmic reticulum,” “cytoplasmic vesicle,” and “ATP-dependent activity.” This enrichment highlights the potential importance of endoplasmic reticulum stress and energy-sensing mechanisms in the comorbid pathways. Notably, the observed enrichment signals for “chromatin organization” and “transcription regulator activity” further imply that upstream epigenetic regulation might synergistically drive the aberrant expression of these immuno-metabolic functional modules. (detailed MendelVar results are presented in S1 Tables [Table 13] and Fig 6).

### 3.9 Cell type-specific expression enrichment analysis

To further investigate the tissue and regulatory specificity underlying the genetic basis of mvIGLMS, transcriptomic and epigenomic data were integrated using the CELLECT framework (detailed CELLECT results are presented in S1 Tables [Table 14]). At the epigenetic level, mvIGLMS-associated genetic loci showed significant enrichment in open chromatin regions and histone modifications across several tissues. Specifically, in pancreatic islets (H3K27ac: *P* = 5.39 × 10^–4^, FDR = 0.0527) and in the fetal adrenal gland (DNase: *P* = 4.02 × 10^–5^, FDR = 0.0197; H3K4me3: *P* = 3.13 × 10^–4^, FDR = 0.0382), enrichment signals were observed. In the dorsolateral prefrontal cortex (DLPFC), a core region for neuro-immune regulation, enrichment was also noted (H3K27ac: **P* *= 2.26 × 10^–4^, FDR = 0.0382; H3K36me3: *P* = 2.31 × 10^–3^, FDR = 0.1129), suggesting a potential link with inflammatory signal transduction. At the transcriptomic level, using GTEx and multi-tissue expression profiles, significant genetic enrichment was clustered primarily in central nervous system regions, particularly within the basal ganglia and frontal cortex (Putamen, basal ganglia: *P* = 0.0101; Caudate, basal ganglia: *P* = 0.0132; Nucleus accumbens, basal ganglia: *P* = 0.0278; frontal cortex (BA9): *P* = 0.0261). Although the FDR correction did not meet the conventional significance threshold (FDR-adjusted *P* > 0.05), these results collectively suggest that the genetic risk for mvIGLMS may be mediated through a brain–gut–endocrine network, potentially acting as a regulatory hub.

### 3.10 Genetic contribution across genomic regions

The conserved regions (e.g., Conserved_LindbladToh.bed) exhibited the highest observed enrichment in genetic contribution across genomic regions (Enrichment = 13.62, FDR = 6.56 × 10^–19^), indicating that highly conserved non-coding sequences likely function as core regulatory elements contributing to cross-phenotype effects. Additionally, several enhancer-associated markers, such as H3K4me1_Trynka (Enrichment = 1.50, FDR = 7.22 × 10^–19^) and H3K9ac_Trynka (Enrichment = 3.69, FDR = 2.38 × 10^–17^), showed significant enrichment across various annotation versions, suggesting that active enhancer regions play an important role in the genetic regulation of IBD-MetS comorbidity through epigenetic mechanisms. These findings collectively suggest that the genetic mechanisms underlying IBD-MetS comorbidity may be largely mediated by conserved transcriptional regulatory regions and enhancer elements, highlighting the potential importance of epigenetic regulation in complex comorbidity networks (S1 Tables [Table 15]).

## 4. Discussion

To the best of our knowledge, this study represents the first systematic analysis of the shared genetic architecture between IBD and MetS. Using genomic SEM we characterized the shared genetic architecture of IBD and MetS. Through integration of six univariate GWAS summary statistics our latent variable model mvIGLMS revealed substantial genetic overlap (CFI = 0.9864; SRMR = 0.0602). In comparison to conventional single trait GWAS, mvIGLMS identified 522 lead SNPs including 21 variants with genome wide significance such as rs12789841, rs1366093, rs159963, rs7193263 and rs7295288. These findings demonstrate the power of multivariate approaches for uncovering cross system genetic effects and provide a novel framework for dissecting molecular mechanisms that link IBD and MetS.

Post-GWAS integration for mvIGLMS highlighted seven genes (*BABAM2*, *ABHD17C*, *SH3YL1*, *HOXC4*, *LPL*, *NMT1*, and *CTSW*) that were significant across multiple analytical platforms (MAGMA, SuSiE, FINEMAP, FUSION, and FOCUS), identifying them as high-confidence genes associated with mvIGLMS. For instance, *HOXC4* may exacerbate autoimmune processes in IBD by upregulating Activation-Induced Cytidine Deaminase (AID) expression, modulating somatic hypermutation and class-switch recombination in B lymphocytes [50–52]. Additionally, differential methylation sites near *HOXC4* (e.g., cg22151644 and cg18473521) are associated with MetS phenotypes, including obesity, T2D, and hypertension [53]. Hypomethylation at these sites could alter *HOXC4* expression, disrupting lipid metabolism or insulin signaling, thereby contributing to MetS. Thus, *HOXC4* may represent a shared molecular basis for both IBD and MetS through its roles in immune regulation (via AID expression) and epigenetic/metabolic signaling (via methylation and TGF-β [Transforming Growth Factor beta] pathways).

*LPL*, a essential enzyme in lipid metabolism, regulates tissue energy balance by hydrolyzing triglycerides in chylomicrons and VLDL, exhibiting tissue-specific metabolic functions in adipose tissue, liver, and immune cells [54]. Dysfunctional *LPL* leads to the accumulation of free fatty acids and oxidized LDL, which activate TLR4/NF-κB and NLRP3 inflammatory pathways, inducing pro-inflammatory cytokine release and establishing a vicious cycle of metabolic and immune dysregulation [55,56]. Consequently, *LPL* may serve as an important link between dysregulated lipid metabolism and chronic inflammation, positioning it as a potential therapeutic target for IBD–MetS comorbidity. Notably, the genes *BRE*, *ABHD17C*, *SH3YL1*, *NMT1*, and *CTSW* have not been previously identified as shared factors in IBD and MetS, warranting further investigation into their roles in mediating common inflammatory and metabolic mechanisms.

In the multivariate genomic SEM model, 21 novel SNP loci were identified that did not achieve significance in univariate GWAS but were significant in mvIGLMS. While these SNPs had not attracted attention within the conventional single-phenotype framework, they showed significant associations with immune cell phenotypes (e.g., eosinophil counts, platelet-to-lymphocyte ratio [PLR]), metabolic biochemical indices (e.g., gamma-glutamyl transferase [GGT], estimated glomerular filtration rate [eGFR]), and neurobehavioral traits (e.g., mood instability, napping behavior) in several external GWAS, suggesting potential pleiotropic effects. Previous studies have not fully characterized these SNPs. For instance, rs11531420, located near the *HTR4* gene, has been linked to Crohn’s disease, and its agonist (e.g., Tegaserod) is used in IBD treatment. It may function by stimulating receptors on intestinal nerve endings to increase neurotransmitter release, thus improving gut motility [57]. Similarly, rs12142313, adjacent to the *MIR181A1HG* gene, could enhance inflammatory signaling by “trapping” the FOXP1 transcription factor, thereby releasing transcriptional inhibition of its target genes [58,59]. Suppression of *MIR181A1HG* reduces monocyte infiltration and plaque formation. Additionally, rs12801000, near the *TNKS1 BP1* gene, modulates lipid metabolism and inflammatory responses by influencing the JAK2/STAT3 pathway. Although primarily studied in the context of liver cancer, its role in lipid metabolism suggests potential involvement in MetS-related pathological processes [60]. While prior research has mostly focused on single-system studies, these genes may exert synergistic effects on IBD and MetS, warranting further exploration.

GSEA and CELLECT analyses uncovered “cross-pathways.” Notably, significant enrichment of gene sets related to neurodevelopment and neurobehavioral traits suggests that the brain–gut–metabolic axis may play a genetic role in the co-occurrence and progression of IBD and metabolic diseases. Genes such as *BDNF* and *CACNA1C*, involved in cognitive and emotional regulation, also play an important roles in the enteric nervous system and metabolic control, demonstrating typical pleiotropic characteristics. At the tissue transcriptomic level, significant genetic enrichment was concentrated in central nervous system regions, including the basal ganglia structures (putamen, caudate, nucleus accumbens) and the frontal cortex. Moreover, the enrichment of gene–environment interaction signals related to lifestyle factors, such as alcohol consumption and smoking, further suggests that the development of IBD and MetS is influenced by genetic factors and enhanced genetic effects under specific environmental exposures. These interactive pathways warrant further validation in real-world cohorts.

Although this study provides novel insights into the genetic basis of mvIGLMS, several limitations remain. Genomic SEM assumes population homogeneity, and the predominance of European-ancestry GWAS summary statistics limits the transferability of findings to other populations. Future research should incorporate more ancestrally diverse samples and population-specific LD references to improve generalizability. Additionally, the shared metabolic basis of certain traits may introduce moderate multicollinearity, potentially affecting the interpretability of factor loadings. To better distinguish overlapping signals, future studies could benefit from more refined phenotypes or more granular modeling approaches. While fine mapping and transcriptomic analyses have identified several genetic loci associated with mvIGLMS, an important challenge remains in linking these loci to specific biological mechanisms. Further investigations should focus on understanding how these genetic variants influence processes such as gene expression and metabolic pathways. The TWAS analysis relied solely on GTEx version 8 reference data, which has limitations in sample size and tissue coverage, potentially limiting the detection of context-specific eQTLs. Incorporating larger, disease-relevant transcriptomic datasets or single-cell eQTL resources would enhance resolution and help validate tissue-specific regulatory mechanisms. Furthermore, while this study highlights the genetic architecture of mvIGLMS, the potential roles of environmental exposures (e.g., smoking, diet, or air pollution) and their interactions with genetic factors require further exploration. Future research should integrate multi-dimensional data to elucidate the gene-environment interplay shaping these phenotypes. Finally, independent replication of the genomic SEM findings was not conducted due to the lack of suitable datasets. Future efforts should include external validation using individual-level or high-quality GWAS summary statistics from independent cohorts.

## 5. Conclusion

By employing a multivariate GWAS framework, the shared genetic architecture between IBD and MetS was systematically dissected. Through genomic SEM, a multivariate model—termed mvIGLMS—was constructed, integrating multiple post-GWAS analytical methods based on the identified shared genetic factors to comprehensively pinpoint potential causal loci. This study offers fresh insights into the genetic regulatory network of mvIGLMS and establishes a foundation for future exploration of intervention targets within the interaction pathways linking IBD and MetS.

## Supporting information

S1 Tables Comprehensive summary of supplementary results. This Excel file provides the complete corresponding analysis results, organized into 15 worksheets [Tables 1–15], each labeled to match the in-text references.
(XLSX)
